# Antioxidant activities of *Sarcodon imbricatum* wildly grown in the Black Sea Region of Turkey

**DOI:** 10.4103/0973-1296.62892

**Published:** 2010-05-05

**Authors:** Tevfik Özen, İbrahim Türkekul

**Affiliations:** *Giresun University, Faculty of Arts and Sciences, Department of Chemistry, 28100 Giresun, Turkey*; 1 *Gaziosmanpaşa University, Faculty of Arts and Sciences, Department of Biology, 60050 Tokat, Turkey*

**Keywords:** Antioxidant activity, chemical content, radical scavenging, *Sarcodon imbricatum*

## Abstract

The antioxidant activities of the methanol extract of *Sarcodon imbricatum* wildly grown in the Black Sea Region of Turkey were investigated in this study. Antioxidant activities were evaluated in terms of total antioxidant activity, reducing power, metal chelating ability, inhibition of linoleic acid peroxidation, superoxide, peroxide and hydrogen peroxide scavenging effects. Various antioxidant activities were compared to references antioxidants such as α-tocopherol, butylated hydroxyanisole (BHA), butylated hydroxytoluene (BHT), and trolox. In total antioxidant (12674.45 μmol α-tocopherol/g of extract), superoxide scavenging (53.74%) and peroxide scavenging activity (45.73%), the methanol extract of *Sarcodon imbricatum* showed stronger activity patterns than that of references antioxidants. Reducing power, metal chelating activity and free radical (DPPH^•^) scavenging activity was increased with the increasing concentration. The contents of total phenolic, flavonoid, anthocyanin, ascorbic acid, β-carotene and lycopene of *Sarcodon imbricatum* were determined and found to be noteworthy.

## INTRODUCTION

Free radicals are described as any chemicals that are able to exist with one or more unpaired outer shell electrons.[1] Reactive oxygen species (ROS) such as superoxide radical (O_2_^−^), hydroxyl radical (OH^−^), singlet oxygen (^1^O_2_) and hydrogen peroxide (H_2_O_2_) are of greatest biological signicance and are generated in a situation of oxidative stress.[2,3] They have an important role related to the pathogenesis of various serious diseases and potentially damaging transient chemical species such as neurodegenerative disorders, cancer, cardiovascular diseases, atherosclerosis, cataracts, and inflammation.[4]

The consumption of plant foods, such as antioxidant supplements or antioxidant-containing foods may be used to protect against various diseases, including cancer, cardio and cerebrovascular diseases. They also help the human body to reduce oxidative damage or protect oxidative deterioration.[5] Antioxidants are the compounds which are synthetic and natural. Natural antioxidants can be phenolic compounds (α-tocopherols, flavonoids, and phenolic acids), nitrogen compounds (alkaloids, chlorophyll derivatives, amino acids, and amines), or carotenoids as well as ascorbic acid, whereas synthetic antioxidants are compounds with phenolic structures of various degrees of alkyl substitution.[6] Synthetic antioxidants such as butylated hydroxyanisole (BHA) and butylated hydroxytoluene (BHT) have been used as antioxidants since the beginning of this century. Restrictions on the use of these compounds, however, are being imposed because of their carcinogenicity and cause an increased interest towards natural antioxidant substances.[7,8] Antioxidant compounds add to food products, especially to lipids and lipid-containing foods and can increase shelf life by retarding the process of lipid peroxidation, which is one of the major reasons for deterioration of food products during processing. An alternative natural and safe source of food antioxidant is found.[9] Thus, the search for natural antioxidants, especially extracted from of plant origin, has notably increased in recent years.[10]

Mushrooms have been used as a food and food flavoring material in soups and sauces for centuries, due to their unique and subtle flavor. They have recently become attractive as functional foods and a source of physiologically beneficial medicine.[11] They contain rich source of phenolic compounds (phenolic acids, flavonoids, carotenoids, α-tocopherol and ascorbic acid).[5,12,13] and there have been several investigations to determine their antioxidant activities based on spectrophotometric techniques in the last years.[13–16]

The medicinal use of mushrooms has a very long tradition in the Asian countries, whereas their use in the Western hemisphere has been slightly increasing only since the last decades.[17] Mushrooms have been used for traditional foods and medicines in Asia.[18] The nutritional values and taste components of commercial mushrooms have been thoroughly investigated.[19] Nevertheless, the edible wild mushrooms are higher prices than cultivated mushrooms. But, people prefer to consume them because of their flavor and texture attributable to their chemical and nutritional properties.[20]

Anatolian people have been using them as food for a long time.[5] Climate and vegetation especially in the northern regions in Turkey are suitable for wild mushroom growing. Especially, the Black Sea Reagion is rainy through the year. *Sarcodon imbricatum*, locally named “*AyІ kulağІ mantarІ*”, is one of the most important common wildly edible mushrooms in Giresun of Turkey. Local people in Giresun of Turkey collect and commonly sell the wild grown *Sarcodon imbricatum* in open market. It ranges from coniferous forest to broad leaves.

In particular, despite widespread consume of *Sarcodon imbricatum* as common-food in Giresun (in the Black Sea Region of Turkey), the literature contains no reports of antioxidant activity and chemical composition of this mushroom. The present study was undertaken to evaluate the antioxidant activity of the methanol extract of *Sarcodon imbricatum* by eight antioxidant assays including total antioxidant activity, inhibition of linoleic acid peroxidation, reducing power, metal chelating, superoxide anion scavenging, free radical scavenging, peroxide scavenging and hydrogen peroxide scavenging activity and to determine its total phenolic compounds, flavonoids, anthocyanins, ascorbic acid, β-carotene and lycopene contents.

## MATERIALS AND METHODS

### Chemicals

Trichloroacetic acid (TCA), Folin-Ciocalteu's reagent, 1,1-diphenyl-2-picryl-hydrazl (DPPH^•^), K_3_Fe(CN)_6_, and ferrous chloride were purchased from E. Merck. Nitroblue tetrazolium (NBT), phenazine methosulphate (PMS), nicotine adenine dinucleotide (NADH), butylated hydroxyanisole (BHA), butylated hydroxytoluene (BHT), α-tocopherol, nicotinamide adenine dinucleotide (NADH), phenazine methosulphate (PMS), potassium ferricyanide and linoleic acid were purchased from Sigma-Aldrich Chemical Co. All other chemicals and reagents were of analytical grade or obtained from Across.

### Mushroom

The *Sarcodon imbricatum* was collected from Giresun (Bektas Plato), in the Black Sea Region of Turkey (Giresun). Identification and classification of macrofungus was carried out and mushroom was deposited at the laboratory of the Department of Chemistry, Giresun University, Giresun, Turkey. Dr. İbrahim Türkekul, Assistant Professor, Department of Biology, Faculty of Arts and Sciences, Gaziosman Paşa University, Tokat, confirmed the taxonomic identity of the mushroom. Fresh mushroom was randomly divided into four samples of 500 g and air-dried in an oven at 40°C. The samples of dried mushroom (10 g) were extracted by stirring with 500 ml of methanol at 30°C at 200 rpm for 24 h filtered with Whatman No. 1 filter paper. The combined methanolic extract was then evaporated at 40°C to dryness, redissolved in methanol to a concentration of 10 mg/ml and stored at 4°C for further use.

### Determination of total phenolic compounds

The total phenolic compounds were determined by Folin Ciocalteu reagent.[21] Briefly, 0.1 ml extract (contains 0.1 mg extract) was mixed with water (46 ml). 1 ml of Folin-Ciocalteu reagent was added. 3 ml of Na_2_CO_3_ (2%) was added. The absorbance of mixture was measured at 760 nm. The standard curve was prepared by 0-100 μg/ml solutions of gallic in ethanol. The concentration of total phenolic compounds in extracts was determined as μg of gallic equivalent using an equation obtained from the standard gallic graph and expressed as mg gallic/g dry weight (DW) of the plant material.

### Determination of total flavonoids

The total flavonoids was determined according to colorimetric method.[22] Briefly, each plant extracts (0.1 g) were dissolved in 1 ml methanol. This solution (0.1 ml) was mixed with 10% AlCl_3_.6H_2_O and 0.1 ml of 1 M potassium acetate (CH_3_COOK). It was kept for 30 min and the absorbance of reaction mixture was measured at 415 nm. Quercetin was chosen as the standard. Using standard curve (0–100 μg/ml), the levels of total flavonoid contents in sample extract were determined in triplicate. The results were calculated into mg quercetin equivalents/g dried plant materials.

### Determination of total anthocyanins

Total anthocyanins were measured according to a slight modification of the methods described earlier.[23] The extracts were mixed with acidified methanol (1% HCl/methanol) for 2 h at 25°C, and then centrifuged at 4000 × g for 10 min. The anthocyanin concentration in the supernatant was measured at 520 and 700 nm, respectively. The absorbance values for 520 and 700 nm were indicated as A_520_ and A_700_, respectively. Following these absorbances of the sample were measured at 520 and 700 nm. Anthocyanins were calculated using the following equation:

Total anthocyanins (mg/ml, Cy-3-glc): (A × MW × DF × 10^3^) / ε × *L*

where A is the absorbance A = (A_520 nm_ − A_700 nm_) _pH 1.0_ − (A_520 nm_ − A_700 nm_) _pH 4.5_, MW is the Cy-3-glc molecular weight: 449.2 g/mol, DF is the dilution factor (0.2 ml sample is diluted to 2 ml, DF = 10), ε is the extinction coefficient (*L* × cm^−1^ × mol^−1^) = 26,900 for Cy-3-glc and *L* (pathlength in cm) = 1.

### Determination of ascorbic acid

Ascorbic acid was determined according to the method of Klein and Perry.[24] Methanolic extract of *Sarcodon imbricatum* was extracted with 10 ml of 1% metaphosphoric acid for 45 min and filtered through Whatman No. 4 filter paper. The filtrate (1 ml) was mixed with 9 ml of 2, 6-dichloroindophenol and the absorbance was measured in 15 s at 515 nm. Content of ascorbic acid was calculated on the basis of the calibration curve of authentic L-ascorbic acid.

### β-carotene and lycopene

β-Carotene and lycopene were determined according to the method of Nagata and Yamashita.[25] The dried methanolic extract (100 mg) was vigorously shaken with 10 ml of acetone-hexane mixture (4:6) for 1 min. The absorbance of the filtrate was measured at 453, 505, 645 and 663 nm. Contents of β-carotene and lycopene were calculated according to the following equations:

lycopene (mg/100 ml) = −0.0458 A_663_ + 0.372 A_505_ − 0.0806 A_453_;

β-carotene (mg/100 ml) = 0.216 A_663_-0.304 A_505_ + 0.452 A_453_.

The results were mean values ± standard deviations. The values were expressed as mg of β-carotene/g of extract and mg of carotenoid/g of extract.

### Determination of total antioxidant activity

The total antioxidant capacity of the crude methanolic extracts of mushroom material was evaluated by the method of Prieto, Pineda, and Aguilar.[26] The antioxidant capacity of the extract was measured spectrophotometrically using a phosphomolybdenum method, based on the reduction of Mo(VI) to Mo(V) by the sample analyte and the subsequent formation of specific green phosphate / Mo(V) compounds. A 0.3 ml aliquot of sample solution (1000 μg/ml) was combined with 2.7 ml of the reagent solution (0.6 M sulfuric acid, 28 mM sodium phosphate and 4 mM ammonium molybdate). The sample was capped and incubated in a boiling water bath at 95°C for 90 min. After the samples had cooled to room temperature, the absorbance was measured at 695 nm. For the blank, 0.3 ml ethanol was mixed with 2.7 ml of the reagent. A typical blank solution contained 2.7 ml of reagent solution and the appropriate volume of methanol used for the dissolution of the samples and it was incubated under the same conditions as the rest of the samples. Stock solutions of α-tocopherol were prepared in methanol just before use. The antioxidant activity of extracts was expressed as equivalents of α-tocopherol using extinction coefficient of 4×10^3^ M^−1^cm^−1^. The total antioxidant activity was expressed as equivalents of α-tocopherol (μmol α-tocopherol /g of extract).

### The inhibition of linoleic acid peroxidation

The procedure was performed according to modified method of Choi *et al*.[27] The extract and antioxidants at the concentration of 100 μg/ml was mixed with linoleic acid solution, in 100 M phosphate buffer pH 7.4, 500 ml of phosphate buffer (100 μM, pH 7.4) and 150 μl of ascorbic acid (10 μM). The linoleic acid peroxidation was initiated by the addition of 0.1 ml FeSO_4_ (10 μM) and incubated at 37°C for 60 min. The mixture of reaction was cooled and added 1.5 ml trichloroacetic acid (10% in 0.5% HCl). Then, 3 ml TBA (1%, in 50 mM NaOH) was added. The reaction mixture and TBA solution were heated in the water bath at 90°C for 60 min. After cooling down, 2 ml aliquots were mixed with 2 ml n-butanol and centrifuged at 1000 × g. The absorbance of upper layer solution was measured spectrophotometrically at 532 nm and calculated the percentage of linoleic acid peroxidation inhibition.

Linoleic acid peroxidation inhibition (%) = [(A_o_-A_1_)/A_o_ × 100],

where A_o_ is the absorbance of control including methanol instead of extracts or standards and A_1_ the absorbance extracts and standards.

### Assay of reducing power

The reducing power of extract was determined according to the method of Oyaizu.[28] *Sarcodon imbricatum* and standard antioxidants (100 μg/ml) in 1 ml of distilled water were mixed with 2.5 ml of phosphate buffer (0.2 M, pH 6.6) and potassium ferricyanide (2.5 ml; 10 g/l). The mixtures were incubated at 50°C for 20 min. Then, a portion of TCA (10%; 2.5 ml) was added to each mixture and centrifuged at 3000 x g for 20 min. Finally, the supernatants (2.5 ml) were mixed with distilled water (2.5 ml) and FeCl_3_ (0.5 ml; 0.1%). The absorbance of the solution was measured at 700 nm.

### Metal chelating activity

The chelating of ferrous ions by extracts was determined by the method of Dinis *et al*.[29] Briefly, the sample (*Sarcodon imbricatum* or antioxidants; 50-500 μg/ml) was added to a solution of 2 mM FeCl_2_ (0.05 ml). The reaction was initiated by the addition of 5 mM ferrozine (0.2 ml) and the mixture was shaken vigorously and left standing at room temperature for 10 min. The absorbance of the resulting solution was then measured at 562 nm. The metal chelating activities were calculated by the given formula:

Metal chelating effect (%) = [(A_0_-A_1_)/A_0_ × 100],

where A_0_ is the absorbance of control and A_1_ is the absorbance of extracts or standards. The control contains FeCl_2_ and ferrozine.

### Assay of superoxide anion scavenging activity

The determination of superoxide anion scavenging activity of extracts was made according to a slightly modified method of Nishimiki *et al*.[30] Superoxide radicals were generated in phenazine methosulfate (PMS)-nicotinamide adenine dinucleotide (NADH) systems by the oxidation of NADH and assayed by the reduction of nitroblue tetrazolium (NBT). One milliter of sample (*Sarcodon imbricatum* or antioxidants; 100 μg/ml), 1.0 ml NBT solution (156 μM NBT in 100 mM phosphate buffer, pH 7.4) and 1.0 ml NADH solution (468 μM in 100 mM phosphate buffer, pH 7.4) were mixed. The reaction was started by adding 100 μl of PMS solution (60 μM PMS in 100 mM phosphate buffer, pH 7.4) to the mixture. The mixture was incubated at 25°C for 5 min, and its absorbance was measured at 560 nm wavelength against blank samples. The decrease absorbance of the mixtures indicates an increasing superoxide anion scavenging activity. The percentage inhibition of superoxide anion generation was calculated using the following formula:

Inhibition of superoxide anion (%) = [(A_0_-A_1_)/A_0_ × 100], where A_0_ is the absorbance of control, and A_1_ is the absorbance of mushroom or standards.

### Assay of free radical scavenging activity

The effect of *Sarcodon imbricatum* on DPPH^•^ radical was estimated according to the method of Blois.[31] wherein the bleaching rate of a stable free radical, DPPH^•^ is monitored at a characteristic wavelength in the presence of sample. An amount of 0.5 ml of 0.1 mM ethanolic solution of DPPH^•^ was added to 3.0 ml of extract or antioxidants solution (100 μg/ml) in water. The mixture was shaken vigorously and kept at room temperature for 30 min. Then the absorbance was measured at 517 nm. The decrease in the absorbance of the DPPH^•^ solution indicates an increasing of DPPH^•^ radical-scavenging activity. This activity was calculated by the following equation:

DPPH^•^ scavenging effect (%) = [(A_0_-A_1_)/A_0_ × 100],

where A_0_ is the absorbance of control and A_1_ is the absorbance of mushrooms or standards.

### Hydrogen peroxide scavenging activity

Hydrogen peroxide scavenging activity was measured according to slightly modified method of Zhao *et al*.[32] 1 ml H_2_O_2_ (0.1 mM) and 1 ml of 100 μg/ml concentration of the extract or standard antioxidants were mixed with 100 l ammonium molybdate (3%), 10 ml H_2_SO_4_ (2 M) and 7 ml KI (1.8 M). The mixed solution was titrated with Na_2_S_2_O_3_ (5 mM) until the yellow color disappears. The percentage scavenging effect was calculated as follows:

Hydrogen peroxide scavenging activity, (%) = (V_o_-V_1_)/V_o_ × 100]

where V_0_ is the volume of Na_2_S_2_O_3_ solution hydrogen peroxide (without extract), V_1_ is the volume of Na_2_S_2_O_3_ solution mixed with the extract or standard antioxidants.

### Peroxide scavenging activity

Peroxide scavenging activity was measured according to a modified method of Smirnoff and Cumbes.[33] Peroxide radicals were generated from the mixture of FeSO_4_ and H_2_O_2_. The reaction mixture contained 1 ml FeSO_4_ (1.5 mM), 0.7 ml H_2_O_2_ (6 mM), 0.3 ml sodium salicylate (20 mM) and sample (extract of *Sarcodon imbricatum* or standards, 100 μg/ml). After incubation for 1 h at 37°C, the absorbance of the hydroxylated salicylate complex was measured at 562 nm. The percentage scavenging effect was calculated as follows:

The peroxide scavenging activity (%)= [1-(A_1_-A_2_)/A_o_] × 100 where A_0_ is the absorbance of the control (without extract or standards) and A_1_ is the absorbance including the extract or standard, A_2_ was the absorbance without sodium salicylate.

### Statistical analysis

For each one of the twelve mushroom species three samples were analyzed and all the determination was carried out in triplicate. The results are expressed as mean values and standard deviation (SD) or standard errors (SE). The results were analyzed using one-way analysis of variance (ANOVA) followed by Tukey's HSD test with α= 0.05. This treatment was carried out using SPSS v.16.0 (Statistical Program for Social Sciences) software.

## RESULTS AND DISCUSSION

### Extractable yield, phenolic compounds, flavonoids, anthocyanins, ascorbic acid, β-carotene and lycopene content

The amount of extractable compounds was 234 mg/g dry plant material for methanol extract of *Sarcodon imbricatum*.

The content of extractable phenolic compounds in extract, determined from regression equation of calibration curve (y=0.3802x+0.0018; *r*^2^=0.9904) and expressed in gallic acid equivalents (GAE). The total phenolic content per gram of crude extract was found 30.20±1.38 mg GAE/g DW. The greater efficiency of methanol in extracting the phenolic compounds would be expected to result in higher antioxidant activity. This value was higher than that reported research.[5–20] The key role of phenolic compounds as scavengers of free radicals is emphasized in several reports.[34,35] The higher content of total phenols in the *Sarcodon imbricatum* extract might account for the better results found in its antioxidant assays.

The total flavonoid content of methanol extract from *Sarcodon imbricatum* extract was assayed by aluminium colorimetric assay as described in Materials and Methods, determined from regression equation of calibration curve (y=3.5721x-0.0027; *r*^2^=0.9961) and expressed in catechin equivalents. The total flavonoid content of methanol extract from *Sarcodon imbricatum* was found 0.260±0.009 mg quercetin/g of DW. Flavonoids are very important plant constituents because of active OH^.−^ and show antioxidant activity.[36]

The content of total anthocyanosides in *Sarcodon imbricatum* extract was 0.13 ± 0.03 as mg cyanidin 3-glucoside/g dry weight. It is to be expected that several activities might be related to a possible antioxidant action from anthocyanosides like polyphenol compounds. The extracts of edible plant were most abundant in phenolics were also most abundant in anthocyanins (which contribute to the total phenolic levels). It has been found that polyphenolic compounds are one of the most effective antioxidant constituents in plant foods, including fruits, vegetables and grains.[37]

The content of ascorbic acid, β-carotene and lycopene in the methanolic extract of *Sarcodon imbricatum* extract was 0.013±0.002 mg ascorbic acid/g DW, 2.08±0.09 μg caratenoid/ g DW, and 2.03±0,05 μg lycopene / g DW, respectively. These values were found in valuable amounts, which were in agreement with other reports concerning ascorbic acid, β-carotene and lycopene quantification in different mushrooms.[11–14]

**Figure 1:**
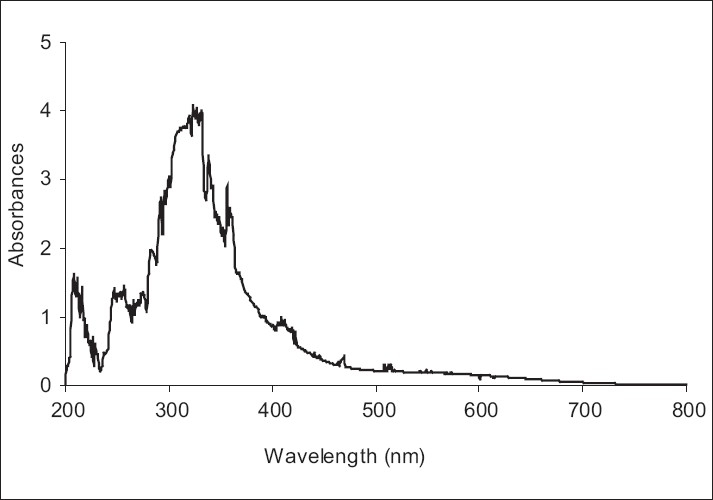
UV-Vis spectra of methanol extract from *Sarcodon imbricatum*

The UV–Vis absorption (200-800 nm) spectra of the mushrooms extracts were assessed for the characterization of phenolic compounds, flavonoid and anthocyanins [Figure 1]. Phenolic compounds which are a first band in the range between 320 and 380 nm and a second band in the 250 to 285 nm range showed two major absorption bands in the UV/Vis regions at 258 (λ_max_), 327 (λ_max_), and 356 (λ_max_).[38,39] λ_max_ of extract 409 and 513 nm may be due to the presence of flavonoids and anthocyanins, respectively. It was found that flavonoids and anthocyanins exhibit comparable special bands to the values reported in the literature.[22–40] These results indicate that the highest content of that phenolic compounds, flavonoids and anthocyanins in the mushroom might contribute to the better results found in their antioxidant activity and found a direct correlation between antioxidant activity *Sarcodon imbricatum* extract and chemical contents.[5–20]

### Total antioxidant activity

The phosphomolybdenum method usually detects antioxidants such as ascorbic acid, some phenolics, α-tocopherol, and carotenoids.[26] The antioxidant activity of the methanol extract of *Sarcodon imbricatum* and references antioxidants at 100 μg/mL concentration as measured by phosphomolybdenum method is presented in Table 1. Total antioxidant activity of *Sarcodon imbricatum* and reference antioxidants exhibited the following order: BHA > *Sarcodon imbricatum* > BHT > trolox. These results were statistically significant, (*P* < 0.05). The results of the three plants are considered to be noteworthy when compared to the findings of other studies concerning medicinal plants in Turkey.[41] The antioxidant activity of mushroom seems to be due to the presence of phenolic compounds, flavonoids and anthocyanosides that may be acted by donating electrons and free radicals.[13–16]

**Table 1:** Total antioxidant activity (μmol α-tocopherol/g of extract), linoleic acid peroxidation (%), superoxide scavenging activity, H_2_O_2_ scavenging activity (%), peroxide scavenging activity (%) of the methanol extract of *Sarcodon imbricatum* at the 100 μg/ml. Values are means ± SD (n=3).

Sample	Total antioxidant activity	Linoleic acid peroxidation	Superoxide scavenging activity	H_2_O_2_ scavenging activity	Peroxide scavenging activity
*Sarcodon imbricatum*	12674.45±16.78	60.57±1.50	53.74±0.07	65.00±2.36	45.73±1.52
α-tocopherol	-	68.43±5.09	44.19±3.57	67.50±1.18	40.45±1.38
BHA	14004.21±65.54	68.64±3.60	48.41±0.25	75.83±1.18	35.81±2.64
BHT	8250.11±14.78	57.63±1.50	45.27±0.11	78.33±7.07	32.92±3.11
Trolox	5692.17±113.08	62.71±4.79	46.81±0.36	72.50±3.54	30.52±1.86

### Inhibition of Linoleic acid peroxidation

Table 1 shows that the extract of *Sarcodon imbricatum* inhibited linoleic acid peroxidation. The results obtained for extract lower than the standards α-tocopherol, BHA, and trolox, but higher than BHT. In conclusion, we can infer that this mushroom extract competes with α-tocopherol, BHA, BHT, and trolox, but not significantly. In previous studies, the inhibition of linoleic acid of methanolic extraction of several commercial and medicinal mushrooms have been reported.[5–42]

### Superoxide scavenging activity

The production of highly reactive oxygen species such as superoxide anion radicals, hydrogen peroxide, and hydroxyl radicals is also catalyzed by free iron through Haber-Weiss reactions.[43] O_2._^−^, which is a reduced form of O_2_, has been implicated in the initiating oxidation reactions associated with aging. In the PMS/NADH-NBT system, superoxide anion derived from dissolved oxygen by PMS/NADH coupling reaction reduces NBT. Antioxidants are able to inhibit the blue NBT formation.[44] The decrease of absorbance at 560 nm with antioxidants thus indicates the consumption of superoxide anion in the reaction mixture. Table 1 presents the superoxide radical scavenging activity of 100 μg/ml mushroom extract in comparison with the same dose of known antioxidants such as α-tocopherol, BHA, BHT, and trolox. *Sarcodon imbricatum* had strong superoxide radical scavenging activity, higher than that of references antioxidants at the same concentrations, (*P* < 0.05). Superoxide radical scavenging activity of those samples followed the order: *Sarcodon imbricatum* > BHA > α-tocopherol > BHA > trolox. The results were found statistically significant (*P*<0.05). It was reported that the superoxide anion scavenging activity could be due to the action of a free hydroxyl group.[45]

### H_2_O_2_ scavenging activity

Table 1 shows that *Sarcodon imbricatum* extract did not show good hydrogen peroxide scavenging activity against H_2_O_2_, the lowest being that of reference antioxidants. H_2_O_2_ scavenging activity of those samples followed the order: BHT > BHA > trolox > α-tocopherol > *Sarcodon imbricatum*. These results were statistically significant, (*P*< 0.05). It is well established that hydrogen peroxide is not dangerous as it is, but may well be because of its ability to form the hydroxyl radical, thereby emphasizing on the importance of its elimination. Indeed, it has previously been proven that dietary phenols protect mammalian and bacterial cells from cytotoxicity induced by hydrogen peroxide,[46] indicating that the observed H_2_O_2_ scavenging activity of our edible mushrooms could be due to the presence of phenols.

### Peroxide scavenging activity

The methanol extract of *Sarcodon imbricatum* had stronger scavenging ability against hydroxyl radicals than the antioxidants (α-tocopherol, BHA, trolox, and BHT) (*P* < 0.05) [Table 1]. Peroxide scavenging effect of extract and standards increased in the order of *Sarcodon imbricatum* > α-tocopherol > BHA > BHT > trolox and these results were statistically significant, (*P*< 0.05). Previous studies had reported two types of antioxidant mechanism: suppression against hydroxyl radical generation, and cleaning hydroxyl radical generated.[47] The former mechanism was related to the transition of metal ions. In the absence of transition metal ions, hydrogen peroxide is fairly stable. However, hydroxyl radicals act in superoxidation by hydrogen peroxide with metal ions, usually ferrous or cupper. The molecules that could chelate iron, and render them inactive of poorly active fenton reaction might have scavenging ability on hydroxyl radical.[48]

### Reducing power

**Figure 2:**
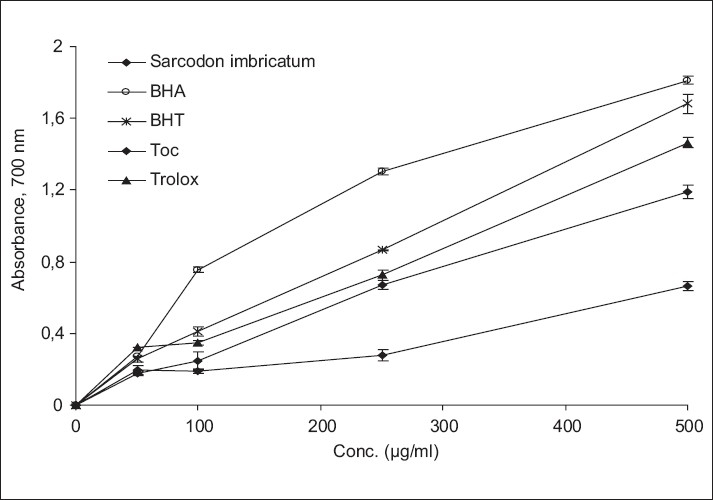
Reducing power of methanol extract from *Sarcodon imbricatum*. α-tocopherol (Toc), BHA, BHT and trolox were used as references antioxidants. Values are means ± SD (n=3).

Figure 2 shows the reducing power of mushroom extract as a function of their concentration (50-500 μg/ml). In this assay, the yellow colour of the test solution changes to various shades of green and blue, depending on the reducing power of each compound.

K3Fe(III)(CN)6+Ph−OH→Kx(Fen(III)Fe(II)(CN)63+Ph−O•+H+

Therefore, measuring the formation of Perl's Prussian blue at 700 nm can monitor the Fe^2+^ concentration.[49] The reducing power of the methanolic extract of *Sarcodon imbricatum* was lower than that of the reference antioxidants. But, the reducing power of the extract increased with concentration significantly, *P*< 0.05. It was reported that the reducing power of mushrooms might be due to their hydrogen-donating ability.[50]

### Metal chelating activity

**Figure 3:**
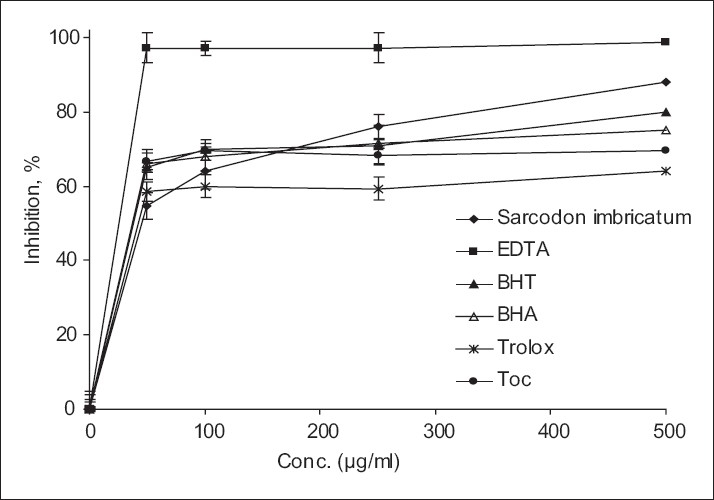
Metal chelating activity of methanol extract from *Sarcodon imbricatum*. α-tocopherol (Toc), BHA, BHT and trolox were used as references antioxidants. Values are means ± SD (n=3).

Iron has the most important lipid pro-oxidant. It is known that Fe^+2^ accelerates lipid peroxidation by breaking down hydrogen and lipid peroxides forms by Fenton free radicallic reaction; $\left({\mathrm{Fe}}^{2+}+H_{2}O_{2}\to {\mathrm{Fe}}^{3+}+{\mathrm{OH}}^{-}+{\mathrm{OH}}^{•}\right)$.[51] Fe^+2^ ion can form complexes with ferrozine. In the presence of chelating agents, the complex formation is prevented, resulting in a decrease in the red color of the complex. Measurement of color reduction allows determination of metal chelating activity. Measurement of the rate of color reduction allows estimation of the chelating activity of the coexisting chelator.[52] In this assay, the methanolic extract of *Sarcodon imbricatum* and the reference antioxidants interfered with the formation of ferrous-ferrozine complex, suggesting that it has chelating activity and captures ferrous ion before ferrozine. Figure 3 shows the chelating effects of the methanolic extract of edible mushroom compared with α-tocopherol, BHA BHT, and trolox as standard on ferrous ions. As can be seen from the figure, chelating capacity of the extracts was increased with the increasing concentration, but not significantly. EDTA was used as a positive control. At a concentration of 500 μg/ml, chelating activity of the extract of mushroom and reference antioxidants were of the following order: EDTA > *Sarcodon imbricatum* > BHT > BHA > α-tocopherol > trolox, with percentage chelations of 98.90, 80.20, 80.00, 75.00, 69.50, and 64,00%, respectively. In parallel to the data obtained from some edible mushrooms that exhibited the methanolic extract of mushroom.[53]

### Free radical (DPPH^•^) scavenging activity

**Figure 4:**
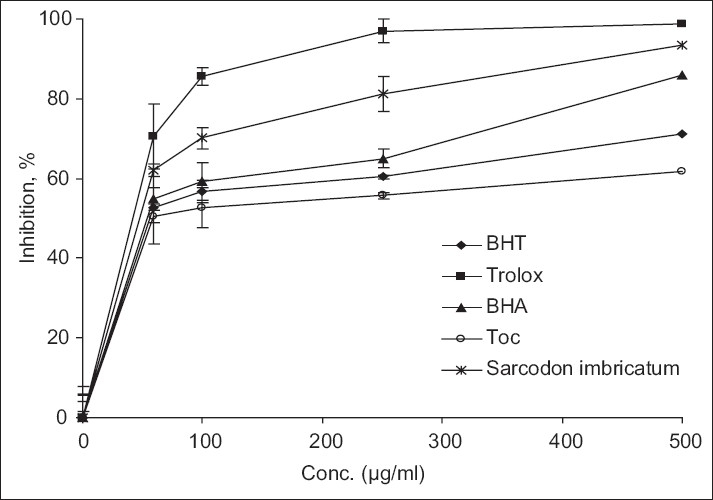
Free radical (DPPH•) scavenging activity of methanol extract from *Sarcodon imbricatum*. α-tocopherol (Toc), BHA, BHT and trolox were used as references antioxidants. Values are means ± SD (n=3).

Free radical scavenging is one of the known mechanisms by which antioxidants inhibit lipid oxidation. The method of scavenging DPPH^•^ free radicals can be used to evaluate the antioxidant activity of specific compounds or extracts in a short time. The radical scavenging of mushroom extract was tested using a methanolic solution of the “stable” free radical, DPPH. *Sarcodon imbricatum* showed appreciable DPPH free radical scavenging activities at the selected scope of concentrations. The methanolic extract of mushroom tested showed much stronger scavenging activities against DPPH compared to antioxidant such as α-tocopherol, BHA, and BHT [Figure 4]. The scavenging effect of *Sarcodon imbricatum* and reference antioxidants on the DPPH· radical were of the following order: trolox > *Sarcodon imbricatum* > BHA > BHT > α-tocopherol, with the percentage scavenging values of 98.80, 93.30, 86.00, 71.30, and 61.65%, respectively, at the concentration of 500 μg/ml. These results were statistically significant, *P*<0.05. The methanolic extracts from other mushrooms showed a moderate increase in DPPH free radical scavenging activities. In the previous studies, the same situations were reported for the methanolic extracts of wild grown mushrooms.[13]

## CONCLUSION

On the basis of the results obtained, we have herein demonstrated the antioxidant activities of the methanol extract of *Sarcodon imbricatum* widely consumed in Giresun of Turkey. The results underline that the extract of mushroom has possessed significant antioxidant properties. Therefore, mushroom extract could be a potential source of natural antioxidants. The consumption of *Sarcodon imbricatum* might give certain level of health protection against oxidative damages. With the established antioxidant activity of this mushroom extract, the specific chemical characteristics and separation of the antioxidative components in the methanol extract should be further investigated.
